# Supplementary material for: Misconception about HIV/AIDS transmission among sexually active women in emerging regions of Ethiopia
Source: PLoS One. 2025 Sep 24;20(9):e0331466. doi: 10.1371/journal.pone.0331466 (PMC12459849; doi:10.1371/journal.pone.0331466)
Supplement: Attachment — Submitted filename: Review of manuscript_PONE-D-24-19228.docx [file pone.0331466.s002.docx]

**Review of manuscript - PONE-D-24-19228**

| **S/N** | **Section** | **Comments/suggestions** |
| --- | --- | --- |
| 1 | Title | 1 and 2 – you need to review the title of your work. It reads “Nearly seven in ten…”, however, your result showed over seven in ten (72.7%). |
| 2 | Abstract | 57 – 58 – how will you increase the awareness of HIV transmission among the poor wealth class household? You need to specify your context: do you think awareness campaigns in these regions will help? |
| 3 | Background | 61 to 68 – Kindly reference the 2023 UNAIDS factsheet which is more accurate and recent. You need to review your grammar, perhaps you can use Grammarly to assist you.  69 to 73 – you should reference 2023 UNAIDS or WHO reports on the mortalities of HIV. This also applies to the data stated between 80 to 84.  91 to 94 – you should state the aim of the study before discussing the public health significance. |
| 4 | Study setting and data source | 107 – women’s record.  110 to 114 – clearly state the two-stage stratified sampling technique.  Did you consider the power of your study when calculating a weighted sample of 497? |
| 5 | Outcome variables | 116 to 117 – Kindly reference the definition of a sexually active reproductive-age woman.  118 – “Yes” to “Health looking person can have HIV/AIDs” is not a misconception!  118 to 121 – you need to reference the source of your questions on misconception. |
| 6 | Data processing and analysis | 133 – summary statistics were presented using “text”? Please, be specific – mean, standard deviation for the quantitative variables, etc.  136 to 139 – the statements are not clear! Kindly review them. |
| 7 | Results |  |
| 8 | Factors associated with the misconception of HIV transmission |  |
| 9 | Tables | Table 1 – kindly reference the categorizations for your Wealth Index.  Table 2 – you have two variables on “Religion”! |
| 10 | Discussion | 197 to 199 – “The probability for high likelihoods of misconception among poorest household wealth categories is due to less or none frequent healthcare visit”. Did you obtain any information on the frequency of hospital visits for this category of participants? Perhaps you should provide another context.  205 – Do you mean Providers Initiated Testing and Counseling (PITC)?  209 to 210 – kindly rephrase the statement. |
| 11 | Strengths and limitations | Could you have used a larger sample size? What was the power of this study? |
| 12 | Conclusion | Kindly look at the conclusion and compare it with your title. |
|  |  |  |
