# Supplementary material for: Misconception about HIV/AIDS transmission among sexually active women in emerging regions of Ethiopia
Source: PLoS One. 2025 Sep 24;20(9):e0331466. doi: 10.1371/journal.pone.0331466 (PMC12459849; doi:10.1371/journal.pone.0331466)
Supplement: Attachment — Submitted filename: Response to reviewers.docx [file pone.0331466.s003.docx]

**Authors’ response to editor and reviewer comments**

We are very grateful to both the editor and reviewers for your comments and suggestions. All the concern raised so far will have an undeniable impact on improving the quality and readability of our scholarly work. Appreciating your effort and valuable comments, we have provided possible reflections and amended the raised concerns and questions. Kindly find our reflections here.

We hope you will consider the revised manuscript acceptable for publication in PLOSE ONE research journal.

| **S.no** | **Editor comments** | **Authors’ responses** |
| --- | --- | --- |
| 1 | Please ensure that your manuscript meets PLOS ONE's style requirements, including those for file naming. The PLOS ONE style templates can be found at  <https://journals.plos.org/plosone/s/file?id=wjVg/PLOSOne_formatting_sample_main_body.pdf> and <https://journals.plos.org/plosone/s/file?id=ba62/PLOSOne_formatting_sample_title_authors_affiliations.pdf> | Dear editor thank you for the comment. We accepted the comments and revised it accordingly. **Kindly check the revised manuscript.** |
| 2 | We notice that your supplementary figures are uploaded with the file type 'Figure'. Please amend the file type to 'Supporting Information'. Please ensure that each Supporting Information file has a legend listed in the manuscript after the references list. | We uploaded the figure as **supporting information** and added a legend at the end of the manuscript after the reference list. Kindly see the revised manuscript on **page 19 line 365-367.** |
| 3 | Please review your reference list to ensure that it is complete and correct. If you have cited papers that have been retracted, please include the rationale for doing so in the manuscript text, or remove these references and replace them with relevant current references. Any changes to the reference list should be mentioned in the rebuttal letter that accompanies your revised manuscript. If you need to cite a retracted article, indicate the article’s retracted status in the References list and also include a citation and full reference for the retraction notice. | We tried to check all the references and we did not used retracted publications. Kindly see the references in the clean version of the manuscript on **page 17-19 line -364.** |
|  | **Reviewer #1:** |  |
| 1 | I have uploaded my review as an attachment. The author needs to review the title and compare it with the results of the study. | Well received for the attachment. We tried to address the title and all your comments in the following section. |
| 2 | Additionally, the author needs to reference UNAIDS/WHO recent report on the burden of HIV. | Dear editor thank you so much. We accepted the comment and referenced the recent UNAIDS/WHO reports. Kindly see **page 4 line 69-71.** |
| 3 | There are several statements that also need to be referenced, this includes the questions that measured the misconception among the study participants. Furthermore, the author needs to the grammar, perhaps Grammarly could be helpful. Other comments will be found in the attached document. | We accepted the comment and added a reference for questions that measured misconceptions. Kindly see the revised manuscript on **page 7 line 123-131.** |
| 4 | Title  1 and 2 – you need to review the title of your work. It reads “**Nearly seven in ten…”, however, your result showed over seven in ten (72.7%).** | Deadly sure. The result depicted more than seven in ten... we revised the title based on reviewer 2 suggestion. We corrected the result section accordingly. Kindly see the revised manuscript on **page 1 line 1-2.** |
| 5 | Abstract  57 – 58 – how will you increase the awareness of HIV transmission among the poor wealth class household? You need to specify your context: do you think awareness campaigns in these regions will help? | Thank you for your question. We add the ways such as extensive health education and campaigns to increase the awareness of HIV transmission. Kindly see the revised manuscript on **page 3 line 56-59.** |
| 6 | Background  61 to 68 – Kindly reference the 2023 UNAIDS factsheet which is more accurate and recent. You need to review your grammar, perhaps you can use Grammarly to assist you. | We accepted the comment and referenced the recent UNAIDS/WHO reports. Kindly see **page 4 line 69-71.** Moreover, we tried to revise the grammar of the whole manuscript. |
| 7 | 69 to 73 – you should reference 2023 UNAIDS or WHO reports on the mortalities of HIV. This also applies to the data stated between 80 to 84. | Dear editor thank you for the comment. We referenced UNAIDS and WHO. Kindly see the revised manuscript on **Page 4 line 75.** |
| 8 | 91 to 94 – you should state the aim of the study before discussing the public health significance. | Thank you for your suggestions. We stated the aim of the study before stating the public health significance. Kindly see the revised manuscript on **page 5 line 93-97.** |
| 9 | **Study setting and data source**  107 – Women’s record. | Thank you for the editing. We accepted the edition and corrected in the manuscript. Kindly see on **page 6 line 109.** |
| 10 | 110 to 114 – clearly state the two-stage stratified sampling technique.  Did you consider the power of your study when calculating a weighted sample of 497? | Dear reviewer, we appreciate your comments. We tried to elaborate the two-stage stratified sampling technique in the revised manuscript on **page 6-7 line 111-121.** Regarding power of our study we mentioned in the limitations section on page **15 line 227-232.** |
| 11 | Outcome variable  116 to 117 – Kindly reference the definition of a sexually active reproductive-age woman. | Dear editor thank you so much for your question. We accepted the comment and added the references based on previous studies. Kindly **refer page 7 line 123-124.** |
| 12 | 118 – “Yes” to “Health looking person can have HIV/AIDs” is not a misconception! | Deadly sure. It is not a misconception. It was tying error. Kindly see on **page 7 line 123-131.** |
| 13 | 118 to 121 – you need to reference the source of your questions on misconception. | Referenced **page 7 line 131.** |
| 14 | **Data processing and analysis**  133 – summary statistics were presented using “text”? Please, be specific – mean, standard deviation for the quantitative variables, etc. | We specified the expressions as frequencies and percentages. Kindly see the revised manuscript on **page 8 line 141.** |
| 15 | 136 to 139 – the statements are not clear! Kindly review them. | Revised on **page 8-9 line 144-149.** |
|  | Results  Factors associated with the misconception of HIV transmission |  |
| 16 | Table 1 – kindly reference the categorizations for your Wealth Index. | We accepted your recommendation and referenced on **Page 8 table 1.** |
| 17 | Table 2 – you have two variables on “Religion”! | Dear editor thank you for your concerns. However, the first one is about religion whereas, the second one is about region. **Kindly see table 2.** |
| 18 | Discussions  197 to 199 – “The probability for high likelihoods of misconception among poorest household wealth categories is due to less or none frequent healthcare visit”. Did you obtain any information on the frequency of hospital visits for this category of participants? Perhaps you should provide another context. | Dear editor thank you so much for your observation. We revised and added another context. Kindly see the revised manuscript on **page 14 line 206-211.** |
| 19 | 205 – Do you mean Providers Initiated Testing and Counseling (PITC)? | Sure. We want to say Providers Initiated Testing and Counseling (PITC). Kindly see the revised manuscript on **page 15 line 215-216.** |
| 20 | 209 to 210 – kindly rephrase the statement. | We appreciate your kind suggestions and rephrased on **Page 15 line 220-221.** |
| 21 | Strength and limitations  Could you have used a larger sample size? What was the power of this study? | We stated in the limitation section. Kindly see on **page 15 line 229-230.** |
| 22 | Conclusion  Kindly look at the conclusion and compare it with your title. | Thank you for your insight. We have seen the conclusion and amended the title. |
|  | **Reviewer # 2** |  |
| 1 | The title requires a re-phrasing to capture of it being a study. Consider for example: "A Study of Misconception about HIV/AIDS among Sexually Active Women in Emerging Region of Ethiopia". | Thank you so much. We accepted and re-phrased the title as your suggestion. Kindly see **page 1 line 1-2.** |
| 2 | There are observed grammatical errors. Line 63: "were died" is incorrect. It should be simply "died" | We accepted your comment and corrected it in the revised manuscript. Kindly see the clean version of the manuscript on **page 4 line 64.** |
| 3 | Line 85 and 86 statement should be re-phrased to convey appropriate message meant to be passed across. Could be re-phrased as: "Despite the country's efforts at reducing the number of HIV cases, it is yet to achieve performance comparable to established objectives" | Dear reviewer, thank you for your comments. We accepted and re-phrased to convey the appropriate message. Kindly see the clean version of the manuscript on **page 5 line 87-88.** |
| 4 | The sentence across Lines 116 and 117 needs to re-phrased for correctness of expression. Consider re-phrasing as: "The outcome variable was contructed by asking sexually active women (at least one sexual outcome in their lifetime) of reproductive age four line item questions | Dear reviewer, thank you for your comments. We accepted and re-phrased to convey the appropriate message. Kindly see the clean version of the manuscript on **page 7 line 123-125** |
| 5 | In list of variables table, the description for "Media Exposure", should entirely be changed to: "Those women who were either reading newspapers/magazine, or listening to radio and watching television at least once a week were considered as having media exposure whereas, those youths who had neither read magazine/newspaper nor listen to radio/ television at all were considered as not having media exposure". | Dear reviewer, thank you for your comments. We accepted and re-phrased to convey the appropriate message. Kindly see the clean version of the manuscript on **page 8 table 1.** |
