# Supplementary material for: Misconception about HIV/AIDS transmission among sexually active women in emerging regions of Ethiopia
Source: PLoS One. 2025 Sep 24;20(9):e0331466. doi: 10.1371/journal.pone.0331466 (PMC12459849; doi:10.1371/journal.pone.0331466)
Supplement: Attachment — Submitted filename: Comments_Manuscript no (PONE-D-24-19228R1).docx [file pone.0331466.s004.docx]

**Abstract**

The definition and the gap of the subject under the study and the title of the research are not matching. As the focus of the study is on misconception, it will be better to describe the background under the abstract about misconception and study gaps related to it.

Better to be consistent when writing phrases and ideas. For instance: Can we use reproductive age women and sexually active women interchangeably? The authors stated…. “Reproductive age women”… in methods, but …”sexually active women…” in result part of the abstract.

Are all reproductive age women sexually active or are all sexually active women in a reproductive age?

**Background**

The authors mentioned about the magnitude and burden of the disease. However, the authors missed to introduce issues related to misconceptions, what types of misconceptions are there currently, evidences misconception and how it results in the raise of HIV/AIDs, magnitude/prevalence, as well as its burden. The authors should also have mentioned any studies previously conducted on misconceptions.

The authors are expected to rewrite the background more about misconception than about the magnitude and burden of the disease (HIV/AIDS) from global to Ethiopia, and clearly state the current gap which leads to misconception.

What is unique among those emerging regions? Do you have evidence that indicates the burden of HIV/AIDs among those emerging regions as compared to other regions? Better to give clue on these issues under the introduction part. What were the gaps of previously conducted studies and what was your plan to fulfil the gap?

**Methods**

What are those 645 enumeration areas? The stratification is by what and how many stratums were formed? The stratification was by what?

For the list of variables in table 1, “distance to the health facility was categorized as “big problem” and “not big problem”. When do we say it is a big problem and when do we say not a big problem? These things have to be mentioned as operational definition or under the outcome variables.

**Results**

The authors need to make the headings of the tables self-explanatory and give important information.

In table 2 for a variable religion, the category give “other” is exceeding an acceptable range or percept, and also what types of religions have been categorized under other should have been mentioned as footnote under the table.

The authors are not consistent in using their variable categories. For instance, in table 2 of their results they mentioned the categories for educational level as “**No education”, “primary” and secondary and higher, however, in table 3, it was mentioned as “unable to read and write”, “primary”, and secondary and higher”. Again, in the table 1, it was mentioned as “No formal education”, “Primary education”, “Secondary and higher education**”.

**Discussion**

The authors should have been discussed how the findings fulfilled the gaps, the importance of their studies. Rather than comparing the finding of the study with other studies done in somewhere, it is better to tell the reader on how their study fulfilled the gap of studies. That will be much more relevant than comparing the finding of the study with the findings of previously conducted studies.

The last paragraph of the discussion (line 215-223), has to be mentioned based on the findings and stated under the recommendation rather than under discussion.
