# Supplementary material for: Misconception about HIV/AIDS transmission among sexually active women in emerging regions of Ethiopia
Source: PLoS One. 2025 Sep 24;20(9):e0331466. doi: 10.1371/journal.pone.0331466 (PMC12459849; doi:10.1371/journal.pone.0331466)
Supplement: Attachment — Submitted filename: Response to reviewers.docxplose.docx [file pone.0331466.s005.docx]

**Authors’ response to editor and reviewer comments**

We are very grateful to both the editor and reviewers for your comments and suggestions. All the concern raised so far will have an undeniable impact on improving the quality and readability of our scholarly work. Appreciating your effort and valuable comments, we have provided possible reflections and amended the raised concerns and questions. Kindly find our reflections here.

We hope you will consider the revised manuscript acceptable for publication in PLOSE ONE research journal.

| **S.no** | **Reviewer 1** | **Authors’ responses** |
| --- | --- | --- |
| 1 | Is it possible to amend the title to ‘Misconception about HIV/AIDS Transmission among Sexually Active Women in Emerging Regions of Ethiopia’? | Thank you. We accepted and revised the title based on your suggestion. **Kindly see page 1 line 1-2.** |
|  | Abstract |  |
| 2 | Methods Indicate the tool used to assess the misconception. | Indicated. Kindly find **page 3 line 47-50.** |
| 3 | Background More than 32 million people died as a result of the disease in the year 2019 [2]. Please use the most recent figure. | Thank you for your comments. We accepted your comment and used the most recent figure. **Page 4 line 66-67.** |
| 4 | The misconception-related evidence should be reported. | Reported in **page 3 line 47-50.** |
| 5 | Policymakers involved in implementing HIV prevention intervention programs will use our findings and valuable resources to combat HIV misconceptions. Better to remove it from the manuscript. | We removed it. Page and line number not applicable |
|  | Methods |  |
| 6 | The study area can be shortened more. | We shortened the study setting. Kindly see Page 7 line 114-115. |
| 7 | Remove ethics approval and informed consent from the manuscript. | We removed it. Page and line number not applicable. |
| 8 | The outcome measure is not indicated. | We tried to indicate the outcome measure. Page 3 line 47-50 and page 7-8 line 134-142. |
| 9 | Results Do you think the Religion frequency is correct? | Yes it is equal frequency with the other variables. We revised the categories. Kindly see page 10 table 2. |
| 10 | What does Distance to the health facility Big problem not problem mean? | The distance to the health facility in DHS is categorized based on women's response to whether it is a big problem or not for the question “how do you perceive distance to the health facility?” |
| 11 | Why misconceptions are dichotomized as yes and no? | We categorized the misconception based on other available literatures. This helps to compare, contrast and discuss with other existing findings. Page 8 line 142. |
| 12 | Please indicate the P value for each result. | We added p value. Kindly see table 3 in the revised manuscript. |
|  | **Reviewer #2:** |  |
| 1 | Abstract The definition and the gap of the subject under the study and the title of the research are not matching. As the focus of the study is on misconception, it will be better to describe the background under the abstract about misconception and study gaps related to it. | We mentioned misconception in the abstract. Kindly see page 1 line 47-50 and page 5 line 89-96. |
| 2 | The authors stated…. “Reproductive age women”… in methods, but …”sexually active women…” in result part of the abstract. | We accepted your comments and made consistent. Kindly see the revised manuscript. |
| 3 | Are all reproductive age women sexually active or are all sexually active women in a reproductive age? | All sexually active women are reproductive age women. We indicated in the manuscript on page 8 line 134-35. |
| 4 | Background The authors mentioned about the magnitude and burden of the disease. However, the authors missed to introduce issues related to misconceptions, what types of misconceptions are there currently, evidences misconception and how it results in the raise of HIV/AIDs, magnitude/prevalence, as well as its burden.  The authors should also have mentioned any studies previously conducted on misconceptions. | Thank you for your comment. We revised the background section. Kindly see on page 5, line 89-96. |
| 5 | The authors are expected to rewrite the background more about misconception than about the magnitude and burden of the disease (HIV/AIDS) from global to Ethiopia, and clearly state the current gap which leads to misconception. | We revised the manuscript on page 5, line 89-96. |
| 6 | What is unique among those emerging regions? Do you have evidence that indicates the burden of HIV/AIDs among those emerging regions as compared to other regions? Better to give clue on these issues under the introduction part. What were the gaps of previously conducted studies and what was your plan to fulfil the gap? | We revised the justification and stated in Page 5 line 100-106. |
| 7 | Methods What are those 645 enumeration areas? The stratification is by what and how many stratums were formed? The stratification was by what? | The Ethiopian Demographic and Health Survey (EDHS) employs a stratified, two-stage cluster sampling design.  1. The primary stratification variable is region, and the secondary stratification is residence. Within each region, EAs are further stratified by urban/rural residence. This creates two sub-strata within each region: urban EAs and rural EAs.  • Within each stratum, a specified number of EAs are selected using probability proportional to size (PPS) sampling. Based on the above approach a total of 645 EAs (202 urban and 443 rural) were used for the survey. The sampling frame for the EDHS is typically based on the most recent Population and Housing Census conducted by the Central Statistical Agency (CSA) of Ethiopia. This census provides a comprehensive listing of enumeration areas (EAs) across the country. |
| 8 | For the list of variables in table 1, “distance to the health facility was categorized as “big problem” and “not big problem”. When do we say it is a big problem and when do we say not a big problem? These things have to be mentioned as operational definition or under the outcome variables. | The distance to the health facility in DHS is categorized based on women's response to whether it is a big problem or not for the question “how do you perceive distance to the health facility?”  We analyzed the available categorized data from the DHS. |
| 9 | Results The authors need to make the headings of the tables self-explanatory and give important information. | We revised the heading of each tables. Kindly see table 1-3. |
| 10 | In table 2 for a variable religion, the category give “other” is exceeding an acceptable range or percept, and also what types of religions have been categorized under other should have been mentioned as footnote under the table | We revised the variable religion. Kindly see table 2. |
| 11 | .The authors are not consistent in using their variable categories. For instance, in table 2 of their results they mentioned the categories for educational level as “No education”, “primary” and secondary and higher, however, in table 3, it was mentioned as “unable to read and write”, “primary”, and secondary and higher”. Again, in the table 1, it was mentioned as “No formal education”, “Primary education”, “Secondary and higher education”. | Thank you for your insight. We revised the category for education. Kindly see the revised manuscript table 2. |
| 11 | Discussion The authors should have been discussed how the findings fulfilled the gaps, the importance of their studies. Rather than comparing the finding of the study with other studies done in somewhere, it is better to tell the reader on how their study fulfilled the gap of studies. That will be much more relevant than comparing the finding of the study with the findings of previously conducted studies | We accepted your comments and revised the discussion. Kindly see the revised manuscript on Page 14 line 191-197. |
| 12 | The last paragraph of the discussion (line 215-223), has to be mentioned based on the findings and stated under the recommendation rather than under discussion. | We moved it to the recommendation section. Kindly see on page 15-16 line 235-240 |
