# Supplementary material for: Misconception about HIV/AIDS transmission among sexually active women in emerging regions of Ethiopia
Source: PLoS One. 2025 Sep 24;20(9):e0331466. doi: 10.1371/journal.pone.0331466 (PMC12459849; doi:10.1371/journal.pone.0331466)
Supplement: Attachment — Submitted filename: Comments.docx [file pone.0331466.s006.docx]

**Comments**

**Abstract**

Background:

- What the authors defined and mentioned as a problem is the magnitude of HIV/AIDS, however, the title of the study is about misconception.
- Better to show that misconception is a problem since it is the subject under the study.

**Methods**

- Better to indicate the result of VIF, to indicate that there is no multi-collinearity.
- Better to include operational definition, and how some variables, for instance “misconceptions on HIV/AIDs”, “sexually active women”

**References**

- Some of the references are written in bold and others are not. So, better to be consistent and impartial across the document.
