# Supplementary material for: Misconception about HIV/AIDS transmission among sexually active women in emerging regions of Ethiopia
Source: PLoS One. 2025 Sep 24;20(9):e0331466. doi: 10.1371/journal.pone.0331466 (PMC12459849; doi:10.1371/journal.pone.0331466)
Supplement: Attachment — Submitted filename: PONE-D-24-19228R2_Review_report_22Jun2025.docx [file pone.0331466.s007.docx]

**PLOS ONE Review Report (*PONE-D-24-19228R2)***

**General comments:**

I appreciated that the authors chose a good topic. However, the documents lack coherence, and needs critical language editions starting from the abstract to recommendation.

**Abstract:**

1. Method is written as if the study was primarily conducted by the authors, which misleads someone who is interested to read the abstract only if get published.

**Background:**

- Line 97-107: There are redundant statements within the same passage.

**Method:**

- Line 124-129: The numbers stated in the passage regarding the sample size, and how it was done is not clear. I think the authors need to be specific about the sample size their paper is concerned with. How did the authors came up with the last sample size (497)?
- Outcome variable: How the study population was operationalized (sexually active women), is not convincing. How can we call a woman is sexually active when she had sex some years back (lifetime), not recently?
- Data processing and analysis (Line 155): It is not clear, which enumeration areas does it refers to. It seems something is lacking.
- Obviously, EDHS data has a hierarchical nature and was already mentioned in the paper. However, the authors did not consider this in analysis, particularly, in the final analysis model.

**Results:**

- The magnitude of misconception seems exaggerated when compared to the national average. It needs to be checked whether how the outcome variable was generated impacted this.

**Discussions:**

- Line 206-207: The authors justified that the discrepancy could be due to the fact other studies are conducted among adolescents. However, the study also included adolescents (15-24years). This seems confusing and need to be discussed in another different way.
- Overall, the paper needs to be critically revised as it currently stands.
