# Supplementary material for: Misconception about HIV/AIDS transmission among sexually active women in emerging regions of Ethiopia
Source: PLoS One. 2025 Sep 24;20(9):e0331466. doi: 10.1371/journal.pone.0331466 (PMC12459849; doi:10.1371/journal.pone.0331466)
Supplement: Attachment — Submitted filename: Response_to_reviewers.docxplose_auresp_3.docx [file pone.0331466.s008.docx]

**Authors’ response to editor and reviewer comments**

We are very grateful to both the editor and reviewers for your comments and suggestions. All the concern raised so far will have an undeniable impact on improving the quality and readability of our scholarly work. Appreciating your effort and valuable comments, we have provided possible reflections and amended the raised concerns and questions. Kindly find our reflections here.

| **S.no** | **Editor comments** | **Authors’ responses** |
| --- | --- | --- |
|  | Abstract |  |
| 1 | Background: What the authors defined and mentioned as a problem is the magnitude of HIV/AIDS, however, the title of the study is about misconception. Better to show that misconception is a problem since it is the subject under the study. | Thank you for this insightful comment. We agree with the reviewer that the problem of misconception about HIV transmission should be more clearly highlighted in the Background section to align with the focus of the study. Accordingly, we have revised the background section of the abstract. Kindly see on **page 3; line 41-44** |
| 2 | Methods: Better to indicate the result of VIF, to indicate that there is no multi-collinearity. | Thank you for the insightful comment. We have now assessed multicollinearity using the Variance Inflation Factor (VIF). Kindly see on **page 9 line 164-166.** |
| 3 | Better to include operational definition, and how some variables, for instance “misconceptions on HIV/AIDs”, “sexually active women” | Thank you for the comment. We have now added operational definitions. Kindly see on **page 7-8 line 131-145**. |
|  | References |  |
| 4 | Some of the references are written in bold and others are not. So, better to be consistent and impartial across the document. | Thank you for pointing this out. We have revised the reference formatting to ensure consistency throughout the document. All references are now uniformly formatted. Kindly see on **page 18 line 280-396.** |
|  | Reviewer 2 |  |
| 1 | General comments:  I appreciated that the authors chose a good topic. However, the documents lack coherence, and needs critical language editions starting from the abstract to recommendation. | Thank you for your constructive feedback. We have thoroughly revised the manuscript for coherence and clarity, from the abstract to the recommendations. Language editing has been applied throughout to improve readability and flow. Kindly see the whole revised manuscript. |
|  | Abstract: |  |
| 2 | Method is written as if the study was primarily conducted by the authors, which misleads someone who is interested to read the abstract only if get published. | Thank you for the important observation. We have revised the Methods section to clearly reflect that the study was a secondary data analysis of the Ethiopian Demographic and Health Survey (EDHS), rather than a primary data collection. Kindly see on **page 3 line 45-47**. |
| 3 | Background:  Line 97-107: There are redundant statements within the same passage. | Thank you for the feedback. We have revised lines 97–107 to remove redundancy and ensure clarity. Kindly see the revised manuscript on **page 5 line 96-105.** |
|  | Method: |  |
| 4 | Line 124-129: The numbers stated in the passage regarding the sample size, and how it was done is not clear. I think the authors need to be specific about the sample size their paper is concerned with. How did the authors came up with the last sample size (497)? | Thank you for the insightful comment. We have revised the section to clarify the sampling process and how the final weighted sample size of 497 was determined. Specifically, we explained that:  A total of 15,683 households were initially included,  From which 3,850 sexually active women were identified,  After removing 633 cases with missing data, 3,217 women remained,  Finally, a weighted sample of 497 sexually active women from emerging regions was analyzed.  This stepwise clarification has now been explicitly stated in the revised Methods section. Kindly see the revised manuscript on **page 6-7 line 122-130.** |
| 5 | Outcome variable: How the study population was operationalized (sexually active women), is not convincing. How can we call a woman is sexually active when she had sex some years back (lifetime), not recently? | Thank you for this important observation. We acknowledge that using “ever had sexual intercourse in a lifetime” as the operational definition of sexually active women may not accurately reflect current sexual activity. This broad definition follows the categorization used in the 2016 Ethiopian Demographic and Health Survey (EDHS), which limits our ability to refine the variable based on recency.  We have now clearly stated this limitation in both the Methods and Limitations sections, explaining that this operationalization may include women not currently sexually active, which could affect the associations observed with misconceptions about HIV transmission. Kindly see on **page 15 line 231-236.** |
| 6 | Data processing and analysis (Line 155): It is not clear, which enumeration areas does it refers to. It seems something is lacking. | Thank you for pointing this out. We have provided additional detail on how EAs were selected using probability proportional to size sampling and their role as clusters in the survey design. This clarification now improves the transparency of the sampling and analysis process. Kindly see on **page 6 line 114-122.** |
| 7 | Obviously, EDHS data has a hierarchical nature and was already mentioned in the paper. However, the authors did not consider this in analysis, particularly, in the final analysis model. | Thank you for this important comment. Although the EDHS data have a hierarchical structure, we conducted an intra-cluster correlation coefficient (ICC) analysis which showed minimal variation between clusters (ICC = 0.012). Additionally, the relatively small sample size per cluster limited the feasibility of multilevel modeling. Therefore, we used a single-level binary logistic regression model for the final analysis. This rationale has now been clearly explained in the Methods and limitations section. Kindly see the revised manuscript on **Page 8-9 line 150-159 and page 16 line 242-246.** |
| 8 | Results:  The magnitude of misconception seems exaggerated when compared to the national average. It needs to be checked whether how the outcome variable was generated impacted this. | Thank you for this valuable observation. We agree that the higher magnitude of misconceptions in our study compared to the national average may be influenced by differences in how the outcome variable was constructed. Our study used a broader definition, including four indicators of misconceptions, while the national estimate was based on fewer items. We have clarified this in the discussion section to explain that the wider definition likely captured more misconceptions, contributing to the higher prevalence observed. Kindly see on **page 14 line 201-210.** |
|  | Discussions: |  |
| 9 | Line 206-207: The authors justified that the discrepancy could be due to the fact other studies are conducted among adolescents. However, the study also included adolescents (15-24years). This seems confusing and need to be discussed in another different way. | Thank you for the helpful comment. We agree that the initial explanation was unclear. We have revised this section to clarify. Kindly see the revised manuscript on **Page 14 line 213-215.** |
| 10 | Overall, the paper needs to be critically revised as it currently stands. | Thank you for your overall feedback. We have carefully revised the entire manuscript to improve clarity, coherence, and academic rigor. Language editing has been applied throughout to enhance readability and flow. We believe these revisions have significantly strengthened the paper. Kindly see the whole revised manuscript. |
